# Preoperative diffusion-weighted magnetic resonance imaging and intraoperative frozen sections for predicting the tumor grade in endometrioid endometrial cancer

**DOI:** 10.18632/oncotarget.26366

**Published:** 2018-11-27

**Authors:** Tomohito Tanaka, Yoshito Terai, Satoe Fujiwara, Yoshimichi Tanaka, Hiroshi Sasaki, Satoshi Tsunetoh, Kazuhiro Yamamoto, Takashi Yamada, Yoshifumi Narumi, Masahide Ohmichi

**Affiliations:** ^1^ Department of Obstetrics and Gynecology, Osaka Medical College, Takatsuki, Japan; ^2^ Department of Obstetrics and Radiology, Osaka Medical College, Takatsuki, Japan; ^3^ Department of Obstetrics and Pathology, Osaka Medical College, Takatsuki, Japan; ^4^ Department of Obstetrics and Gynecology, First Towakai Hospital, Takatsuki, Japan; ^5^ Department of Obstetrics and Radiology, First Towakai Hospital, Takatsuki, Japan

**Keywords:** endometrial cancer, diffusion weighted image, apparent diffusion coefficient, frozen section, grade

## Abstract

**Objective:**

The histological tumor grade is a strong predictor of nodal metastasis in endometrial cancer; as such, an accurate pre- or intraoperative diagnosis is important for performing lymphadenectomy.

**Methods:**

Ninety-one patients with endometrioid endometrial cancer were imaged on DW-MRI with the apparent diffusion coefficient (ADC) calculated and a frozen section (FS) diagnosis made before and at hysterectomy. The diagnostic accuracy for predicting the tumor grade for diffusion weighted magnetic resonance inaging (DW-MRI) and the FS diagnosis compared to the ultimate histologic status was analyzed.

**Results:**

Among 91 patients with endometrioid endometrial cancer, high-grade (endometrioid G3) tumors had lower ADC values than low-grade (endometrioid G1/2) tumors. The cut-off of the mean ADC_mean_ values for predicting high-grade tumors resulted in 743×10^-6^ mm^2^/sec according to the receiver operating characteristic curve. The true positive rates of ADC values and FSs for the prediction of high-grade tumors did not differ to a statistically significant extent (73.3% vs. 66.7%, p=0.7), however, the true negative rate of ADC values for the prediction of low-grade tumors was significantly lower than that of the FSs (64.5% vs. 98.7%, p=0.01). The kappa statistics of ADC values and FSs were 0.23 and 0.73, respectively. Of note, all five patients with high-grade tumors for whom intraoperative FSs indicated low-grade tumors were predicted to have high-grade tumors on preoperative DW-MRI.

**Conclusion:**

A FS diagnosis is more suitable for predicting high-grade tumors than DW-MRI; however, physicians should pay close attention to tumors with low ADC values on preoperative DW-MRI.

## INTRODUCTION

The histological tumor grade is an important factor for predicting lymph node metastasis and the prognosis in endometrial cancer. In patients with endometrioid endometrial cancer, the rate of lymph node metastasis was 2.8% in grade 1 tumors and 5.7% in grade 2 tumors but increased to 9.4% in patents with grade 3 tumors [1]. Pelvic lymph node dissection (PLND) remains an important surgical procedure for treating endometrial cancer. This procedure is necessary to obtain correct staging and has resulted in a favorable prognosis, especially in patients with high-risk endometrial cancer [2–10], however it may not be needed for low-risk cases, including cases involving low-grade tumors and cases without myometrial invasion. Therefore, the histological tumor grade is an important factor for making decisions regarding surgical procedures, including lymph node dissection. Generally, the tumor grade is determined with a preoperative biopsy [11].

Magnetic resonance imaging (MRI) has been shown to be an accurate imaging technique for the preoperative assessment of endometrial cancer and for evaluating the depth of myometrial invasion [11]. Diffusion-weighted MRI (DW-MRI) is a useful imaging technique for evaluating the Brownian motion of water in tissues. In biological tissues, this motion is restricted by interactions with cell membranes and macromolecules on a microscopic level. Increased tissue cellularity, as seen in tumors, restricts Brownian motion, which can be quantified by the calculation of the apparent diffusion coefficient (ADC).

The purpose of this study was to determine if there is a correlation between the histological tumor grade and ADC value and to evaluate the diagnostic accuracy of the tumor grade for preoperative DW-MRI and intraoperative frozen sections (FSs) in endometrioid endometrial cancer patients.

## RESULTS

### Characteristics of the study participants in test and validation set

A total of 109 patients with endometrioid endometrial cancer underwent preoperative DW-MRI, preoperative endometrial biopsy and the intraoperative examination of FSs. Ninety-one patients were recruited in the test set. The mean age of the patients was 57.9 ± 10.8 years, and the mean body mass index (BMI) was 23.9 ± 4.2. According to the histopathological diagnosis of surgical specimens, a total of 66 (72.5%) patients had International Federation of Gynecology and Obstetrics (FIGO) stage IA disease, 12 (13.2%) had stage IB disease, 1 (1.1%) had stage II disease, 6 (6.6%) had stage IIIA disease, 3 (3.3%) had stage IIIC disease and 3 (3.3%) had stage IV disease. Histologically, 58 (63.7%) patients had endometrioid carcinoma of grade 1, 18 (19.8%) had grade 2, and 15 (16.5%) had grade 3. Eighteen patients were recruited in the validation set. The mean age of the patients was 57.2 ± 11.3 years, and the mean BMI was 22.3 ± 4.8. A total of 11 (61.1%) patients had FIGO stage IA disease and 7 (38.9%) had stage IB disease. Histologically, 11 (61.1%) patients had endometrioid carcinoma of grade 1, 5 (27.8%) had grade 2, and 2 (11.1%) had grade 3 (Table 1).

**Table 1:** Characteristics of patients with endometrial cancer who underwent pre-operative DW-MRI and intra-operative frozen section analyses

Characteristic	Test set (%)	Validation set (%)
Number of patients	91	18
Age, years old^*^	57.9 ± 10.8	57.2 ± 11.3
BMI, kg/m^2*^	23.9 ± 4.2	22.3 ± 4.8
FIGO stage		
IA	66 (72.5)	11 (61.1)
IB	12 (13.2)	7 (38.9)
II	1 (1.1)	0
IIIA	6 (6.6)	0
IIIC	3 (3.3)	0
IV	3 (3.3)	0
Grade		
1	58 (63.7)	11 (61.1)
2	18 (19.8)	5 (27.8)
3	15 (16.5)	2 (11.1)

*Based on an ANOVA (mean ± SD)

Abbreviations: BMI, body mass index; FIGO, federation of Gynecology and Obstetrics.

### The ADC values for each tumor grade in the test set

**Figure 1:**
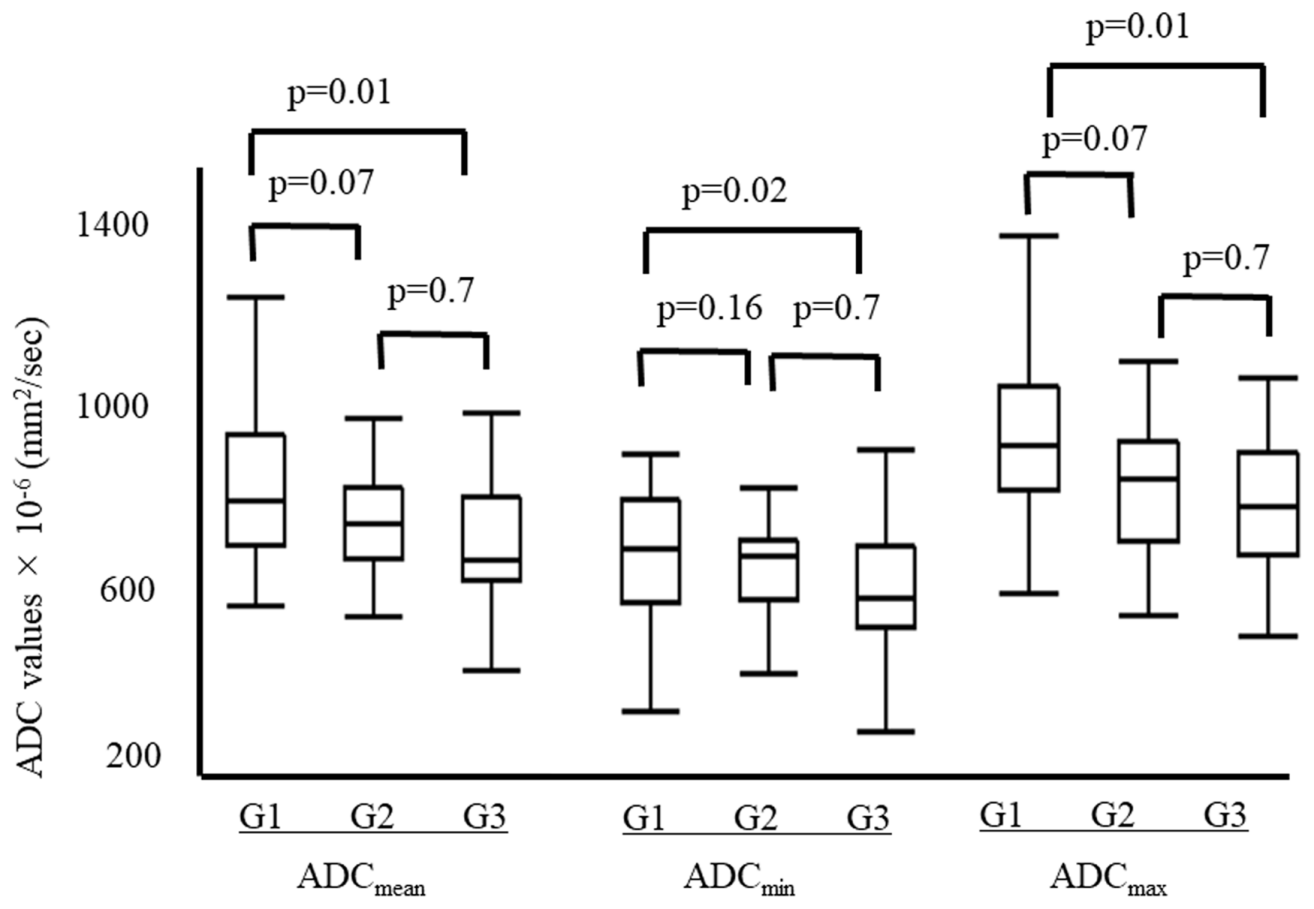
The ADC values for each tumor grade The mean ADC_mean_ was 882 ± 264 × 10^-6^ (mm^2^/sec) for grade 1 tumors, 745 ± 115 × 10^-6^ (mm^2^/sec) for grade 2 tumors and 686 ± 149 × 10^-6^ (mm^2^/sec) for grade 3 tumors. The mean ADC_min_ was 737 ± 224 × 10^-6^ (mm^2^/sec) for grade 1 tumors, 640 ± 103 × 10^-6^ (mm^2^/sec) for grade 2 tumors and 586 ± 149 × 10^-6^ (mm^2^/sec) for grade 3 tumors. The mean ADC_max_ was 1048 ± 306 × 10^-6^ (mm^2^/sec) for grade 1 tumors, 883 ± 207 × 10^-6^ (mm^2^/sec) for grade 2 tumors and 813 ± 168 × 10^-6^ (mm^2^/sec) for grade 3 tumors. Each of the mean ADC values was significantly higher for grade 1 tumors than for grade 3 tumors. In contrast, none of the mean ADC values differed significantly between grade 1 and 2 tumors and grade 2 and 3 tumors.

Among the 91 patients in test set, the mean ADC_mean_ was 882 ± 264 × 10^-6^ (mm^2^/sec) for grade 1 tumors, 745 ± 115 × 10^-6^ (mm^2^/sec) for grade 2 tumors and 686 ± 149 × 10^-6^ (mm^2^/sec) for grade 3 tumors. The mean ADC_mim_ was 737 ± 224 × 10^-6^ (mm^2^/sec) for grade 1 tumors, 640 ± 103 × 10^-6^ (mm^2^/sec) for grade 2 tumors and 586 ± 149 × 10^-6^ (mm^2^/sec) for grade 3 tumors. The mean ADC_max_ was 1048 ± 306 × 10^-6^ (mm^2^/sec) for grade 1 tumors, 883 ± 207 × 10^-6^ (mm^2^/sec) for grade 2 tumors and 813 ± 168 × 10^-6^ (mm^2^/sec) for grade 3 tumors. Each of the three ADC values was significantly higher for grade 1 tumors than for grade 3 tumors. In contrast, the ADC values were not different significantly between grade 1 and 2 tumors and between grade 2 and 3 tumors (Figure 1).

### The diagnostic accuracy of the ADC values, preoperative biopsy and intraoperative FSs in predicting high-grade tumors in the test set

**Figure 2:**
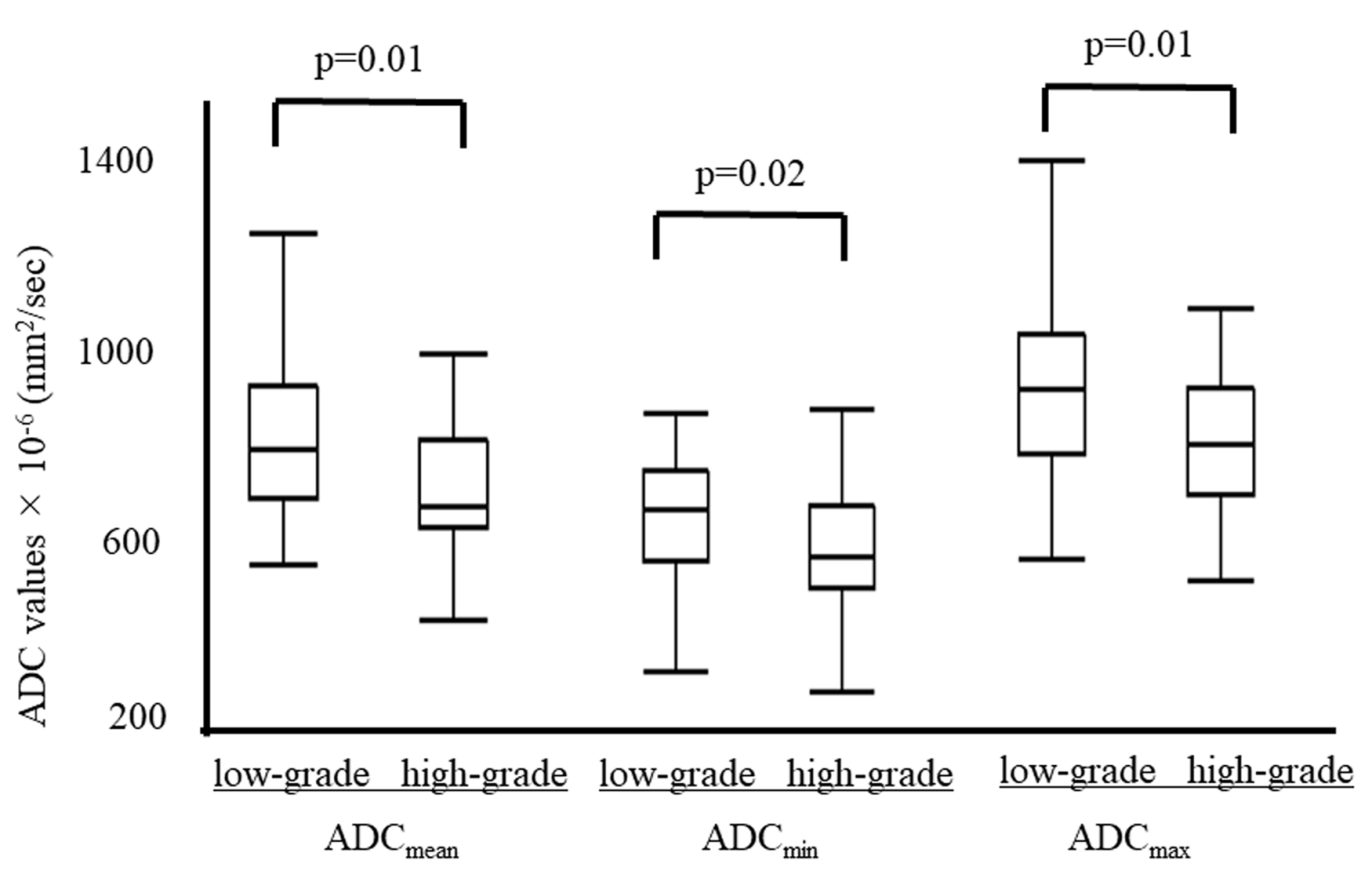
The ADC values compared between low-grade (grade 1/2) and high-grade (grade 3) tumors The mean ADC_mean_, ADC_min_ and ADC_max_ were significantly lower in high-grade tumors than in low-grade tumors (ADC_mean_, 686 ± 149 vs. 850 ± 244 × 10^-6^ mm^2^/sec, p=0.01; ADC_min_, 586 ± 149 vs. 714 ± 206 × 10^-6^ mm^2^/sec, p=0.02; ADC_max_, 1009 ± 293 vs. 814 ± 168 × 10^-6^ mm^2^/sec, p=0.01).

**Figure 3:**
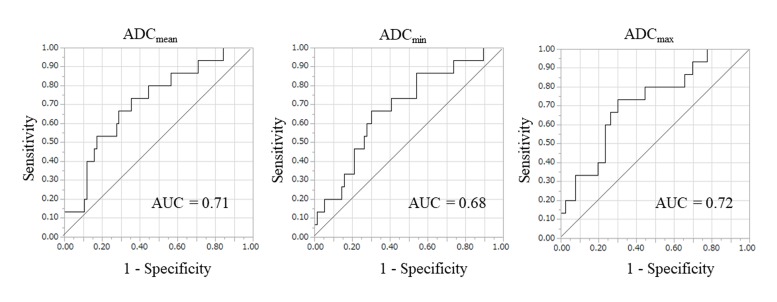
The receiver operating characteristic curve (ROC) for predicting high-grade tumors The area under curve (AUC) values of the ADC_mean_, ADC_min_ and ADC_max_ for predicting high-grade tumors were 0.71, 0.68 and 0.72, respectively. According to ROC analysis, the cut-off values of the ADC_mean_, ADC_min_ and ADC_max_ for predicting high-grade tumors were 743, 603 and 877 × 10^-6^ (mm^2^/s), respectively.

Among 91 patients in test set, the mean ADC_mean_, ADC_min_ and ADC_max_ were significantly lower in the high-grade tumors than in the low-grade tumors (ADC_mean_, 686 ± 149 vs. 850 ± 244 × 10^-6^ mm^2^/sec, p=0.01; ADC_min_, 586 ± 149 vs. 714 ± 206 × 10^-6^ mm^2^/sec, p=0.02; ADC_max_, 1009 ± 293 vs. 814 ± 168 × 10^-6^ mm^2^/sec, p=0.01) (Figure 2). The area under the curve (AUC) values of the ADC_mean_, ADC_min_ and ADC_max_ for predicting high-grade tumors were 0.71, 0.68 and 0.72, respectively. Figure 3 shows the receiver operating characteristic curves (ROCs). The cut-off values of ADC_mean_, ADC_min_ and ADC_max_ for predicting high-grade tumors were 743, 603 and 877 × 10^-6^ (mm^2^/sec), respectively.

The true positive rates of ADC_mean_, ADC_min_, ADC_max_, preoperative biopsy, and FS in the diagnosis of high-grade rumors were 73.3%, 66.7%, 73.3%, 40.0% and 66.7%, respectively. The true negative rates of ADC_mean_, ADC_min_, ADC_max_, preoperative biopsy, and FS in the diagnosis of low-grade tumors were 64.5%, 69.7%, 69.7%, 89.0% and 98.7%, respectively. The kappa statistics (95% confidence interval) of ADC_mean_, ADC_min_, ADC_max_, preoperative biopsy, and FS were 0.23 (0.06-0.41), 0.25 (0.05-0.44), 0.29 (0.10-0.47), 0.43 (0.17-0.69) and 0.73 (0.53-0.93), respectively (Table 2). The true positive rates of combined methods in the prediction of high-grade tumors were as follows: preoperative biopsy and FS, 73.3%; FS and DW-MRI, 100%; and preoperative biopsy and DW-MRI, 80%. Although the combination of FS and DW-MRI was had a true positive rate of 100%, the true negative rate of this combination in the prediction of low-grade tumors was 63.2% with a kappa statistic of 0.36. Thus, the combination of FS and DW-MRI had an excellent true positive rate for the prediction high-grade tumors, but was associated with a high rate of false positive results. Of note, all five patients with high-grade tumors for whom intraoperative frozen sections indicated low-grade tumors were predicted to have high-grade tumors on preoperative DW-MRI. On preoperative endometrial biopsy, the diagnoses of these 5 cases were endometrioid carcinoma G1 (n=2), G2 (n=2) and G3 (n=1).

**Table 2:** The correlation of the tumor grade with the preoperative MRI parameters, and the preoperative biopsy, intraoperative frozen section and final paraffin section findings in the test set

	ADCmean		ADCmin		ADCmax		Biopsy		FS		PS
	G1 or G2	G3	G1 or G2	G3	G1 or G2	G3	G1 or G2	G3	G1 or G2	G3	
G1 or G2	49	27	53	23	53	23	73	3	75	1	76 (83.5)
G3	4	11	5	10	4	11	9	6	5	10	15 (16.5)
Total	53	38	58	33	57	34	82	9	80	11	91
True positive high-grade tumors	73.3%		66.7%		73.3%		40.0%		66.7%		
True negative low-grade tumors	64.5%		69.7%		69.7%		89.0%		98.7%		
Kappa statistics (95% CI)	0.23 (0.06-0.41)		0.25 (0.05-0.44)		0.29 (0.10-0.47)		0.43 (0.17-0.69)		0.73 (0.53-0.93)		

Abbreviations: ADC, apparent diffusion coefficient; FS, frozen section; PS, paraffin section.

### The ADC values of low-grade (grade 1/2) and high-grade (grade 3) tumors in the validation set

When the cutoff value in test set was applied to the 18 patients in validation set, the true positive rates of the ADC_mean_, ADC_min_ and ADC_max_ in the prediction of high-grade tumors were 100%, 50.0% and 50.0%, respectively. The true negative rates of the ADC_mean_, ADC_min_, and ADC_max_ in the prediction of low-grade tumors were 68.8%, 75.0% and 81.3%, respectively. The kappa statistics of the ADC_mean_, ADC_min_ and ADC_max_ were 0.33, 0.15 and 0.22, respectively (Table 3).

**Table 3:** The correlation of the tumor grade with the preoperative MRI parameters and the final paraffin section findings in the validation set

	ADCmean		ADCmin		ADCmax		PS
	G1 or G2	G3	G1 or G2	G3	G1 or G2	G3	
G1 or G2	11	5	12	4	13	3	16 (88.9)
G3	0	2	1	1	1	1	2 (11.1)
Total	11	7	13	5	14	4	18
True positive high-grade tumors	100%		50.0%		50.0%		
True negative low-grade tumors	68.8%		75.0%		81.3%		
Kappa statistics	0.33		0.15		0.22		

Abbreviations: ADC, apparent diffusion coefficient; FS, frozen section; PS, paraffin section.

## DISCUSSION

In the current study, the true positive rates of the ADC values and FSs in the prediction of high-grade tumors did not differ to a statistically significant extent; however, the true negative rate of the ADC values in the prediction of low-grade tumors was significantly lower than that of FSs. The kappa statistics of the ADC value and FS were 0.23 and 0.73, respectively. Although FSs were found to be more useful for predicting high-grade tumors than DW-MRI, all five patients with high-grade tumors for whom intraoperative frozen sections indicated low-grade tumors were predicted to have high-grade tumors on preoperative DW-MRI. Therefore, careful management should be performed in cases of endometrioid tumors with low ADC values.

While the ADC values of endometrial cancer have been evaluated, the relationship between the ADC values and histological grade remains controversial. Several authors found no correlation with the tumor grade [12–14], whereas more recent studies have shown that high-grade tumors have lower ADC values than low-grade tumors [15–17]. Rechichi et al. reported a prospective study of ADC values in endometrial cancer. Among 106 patients, including 70 endometrial cancer patients and 36 control subjects with normal endometrium, the mean ADC value of endometrial cancer tissue was significantly lower than that of normal endometrium; however, there was no significant difference in the ADC values between the histological grade. In this study, the mean ADC values (×10^-6^ mm^2^/sec) of grade 1, 2 and 3 tumors were 790 ± 8, 760 ± 14 and 750 ± 12, respectively [12]. Bharwani et al. reported the ADC values in 42 endometrial lesions, including 23 endometrial cancers and 19 benign lesions. Although the mean ADC value for endometrial carcinoma tissue was significantly lower than that of benign endometrial tissue, there were no statistically significant differences between the tumor grades. The cut-off of the mean ADC values for predicting malignancy was <1280 × 10^-6^ (mm^2^/sec). In this setting, the sensitivity and specificity for malignancy were 87% and 100%, respectively. The mean and minimum ADC values (×10^-6^ mm^2^/sec) for each histological grade were 1020 ± 290 and 740 ± 240 (grade 1), 880 ± 390 and 640 ± 360 (grade 2) and 940 ± 320 and 720 ± 360 (grade 3), respectively [13]. Kishimoto et al. showed that the ADC values were significantly inversely correlated with the tumor cellularity. However, no significant relationship was observed between the ADC values and tumor grade. The mean ADC values (×10^-6^ mm^2^/sec) of grade 1, 2 and 3 tumors were 880 ± 265, 800 ± 178 and 810 ± 117, respectively [14]. Woo et al. reported a histogram analysis of ADC values based on the entire tumor volume to determine the grade of endometrial cancer in 33 patients. The standard deviation, quartile and 75^th^, 90^th^ and 95^th^ percentiles of ADC showed significant differences between grades and between high and low grades. An ROC curve analysis yielded sensitivities and specificities of 75% and 96%, 62.5% and 92%, 100% and 52%, 100% and 72%, and 100% and 80%, using the standard deviation, quartiles and 75^th^, 90^th^ and 95^th^ percentiles to identify high-grade tumors with corresponding areas under the curve of 787, 792, 765 880 and 925 × 10^-6^ (mm^2^/sec), respectively [15]. Nougaret et al. showed that the minimum 10^th^, 25^th^, 50^th^, 75^th^ and 90^th^ percentile ADC values were significantly lower in grade 3 tumors than in grade 1 and 2 tumors among 97 endometrial cancer patients. For the medium ADC values according to the ROC curve results, an ADC value lower than 730 × 10^-6^ (mm^2^/sec) was associated with grade 3 tumors with a sensitivity of 77% and specificity of 75% [16]. Yan et al. reported the ADC values in 98 endometrial cancer patients. The mean ADC values for high-grade endometrioid adenocarcinomas were significantly lower than those for low-grade tumors (800 vs. 962 × 10^-6^ [mm^2^/sec], p=0.002). However, no significant differences were noted in mean and minimum ADC values among tumor grades [17]. Although the relationship between the ADC values and histological tumor grade remains controversial, grade 3 tumors have lower ADC value than grade 1 tumors in many published literatures. In the current study, the ADC values were significantly lower for grade 3 tumors than for grade 1 tumors.

In contrast, it has been reported that intraoperative FS diagnoses tend to agree with the final pathology [11, 18, 19]. Karabagli et al. reported that the agreement for tumor grade between FSs and PSs was 89.9%, with a kappa value of 0.84 [18]. Stephan et al. reported that the histological grade evaluation based on an FS had 98% sensitivity and 53% specificity compared to PSs. There were no discrepancies in the FS assessment of high-grade tumors, with all confirmed by PSs [19]. We previously reported on the histological agreement between FSs and PSs in endometrial cancer. When grade 3 endometrioid carcinoma, serous carcinoma, clear cell carcinoma and carcinosarcoma were considered high-grade tumors, the grade evaluation at the time of obtaining an FS had 70.2% sensitivity and 99.0% specificity, with a kappa value of 0.75 [11].

The present study is associated with three major limitations that may reduce its value. First, the sample size was quite small for assessing the apparent ADC values. Second, the same pathologists performed both the FS and PS evaluations; this may have caused some bias in favor of the FS diagnosis. Third, the ADC values differed among tumor sites. Fourth, the fact that the whole tumor was not defined and that the ADC_mean_, ADC_min_ and ADC_max_ were reported might have influenced the outcome. As such, our results must be confirmed in further studies.

In conclusion, true positive rates of ADC values and frozen sections in the prediction of high-grade tumors did not differ to a statistically significant extent, however, the true negative rate of the ADC values in the prediction of low-grade tumors was significantly lower than that of FSs; DW-MRI had an excellent true positive rate for the prediction high-grade tumors, but was associated with a high rate of false positive results. Although FSs were found to be more useful for predicting high-grade tumors than DW-MRI, all five patients with high-grade tumors for whom intraoperative frozen sections indicated low-grade tumors were predicted to have high-grade tumors on preoperative DW-MRI. Given these findings, careful management should be performed for endometrioid tumors with low ADC values.

## MATERIALS AND METHODS

### Participants

Between September 2014 and July 2018, a total of 109 patients with a diagnosis based on preoperative endometrial curettage, preoperative MRI and intraoperative FSs underwent hysterectomy for endometrial cancer at Osaka Medical College in Japan. Patients who met the following criteria were eligible for inclusion in the study: (1) underwent preoperative DW-MRI with ADC map at First Towakai hospital by the same radiologist with the same imaging systems; (2) underwent laparoscopic or abdominal hysterectomy with bilateral salpingo-oophorectomy at Osaka Medical College; (3) received an intraoperative FS diagnosis; (4) did not receive any treatment, including chemotherapy and radiotherapy, before surgery. Ninety-one patients who were treated between September 2014 and September 2017 were assigned to the test set and 18 patients treated between October 2017 and July 2018 were assigned to the validation set.

After obtaining Institutional Review Board approval, the medical records were retrospectively reviewed. The ADC values on DW-MRI were evaluated in each tumor grade and the final determinations were made based on the findings of evaluations using paraffin sections (PSs). The histological grades determined based on preoperative DW-MRI or FS findings and on PS were also assessed.

### MRI

All pelvic MR images were obtained using a 1.5-T MR imaging unit (Philips Healthcare, Best, The Netherlands) within a month before surgery. Routine pelvic MRI scans were acquired as Supplementary Table 1. Axial oblique scans were obtained perpendicular to the endometrial cavity, resulting in a short-axis view. Dynamic contrast material-enhanced (DCE) imaging of the pelvis was performed after the administration of 0.1 mmol/kg of body weight gadolinium chelate (Gadovist; Bayer, Mississauga, Ontario, Canada) using 3-dimensional gradient-echo T1 weighted SPAIR with the parameters in Supplementary Table 1. Images were acquired at multiple phases of contrast medium enhancement in both the sagittal and axial oblique planes at 30, 60, 90 and 180 seconds.

### ADC analyses of endometrial lesions

**Figure 4:**
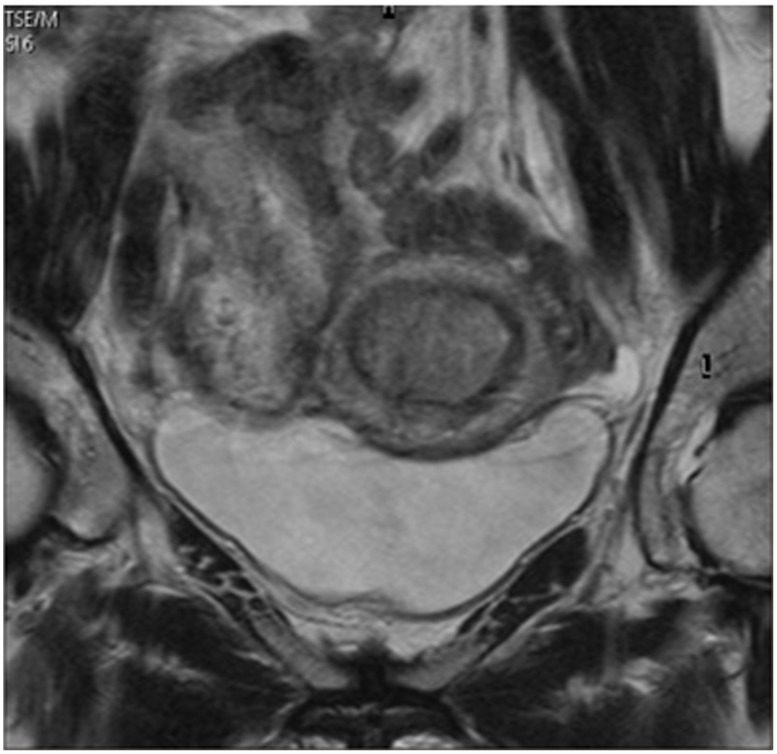

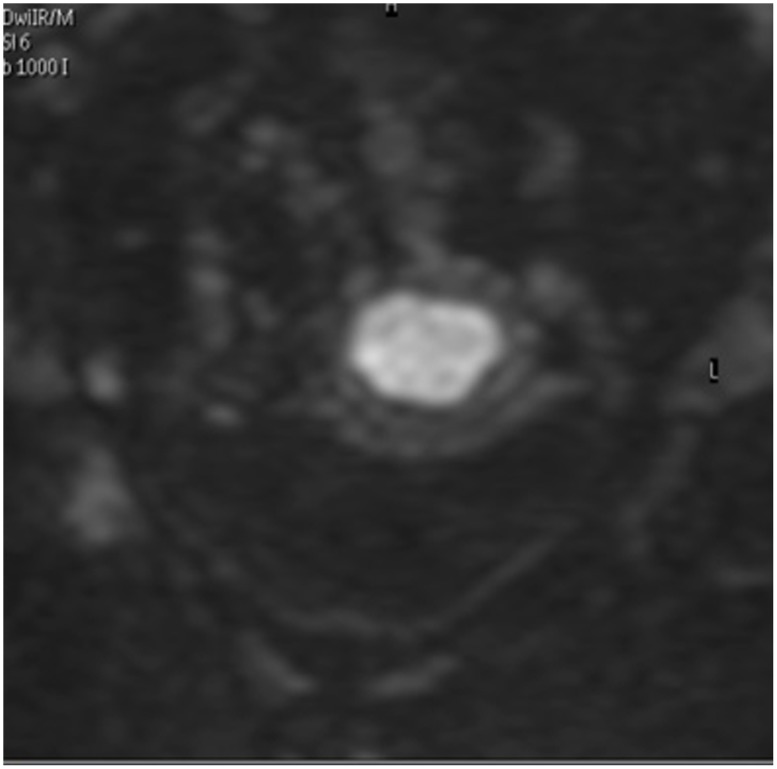

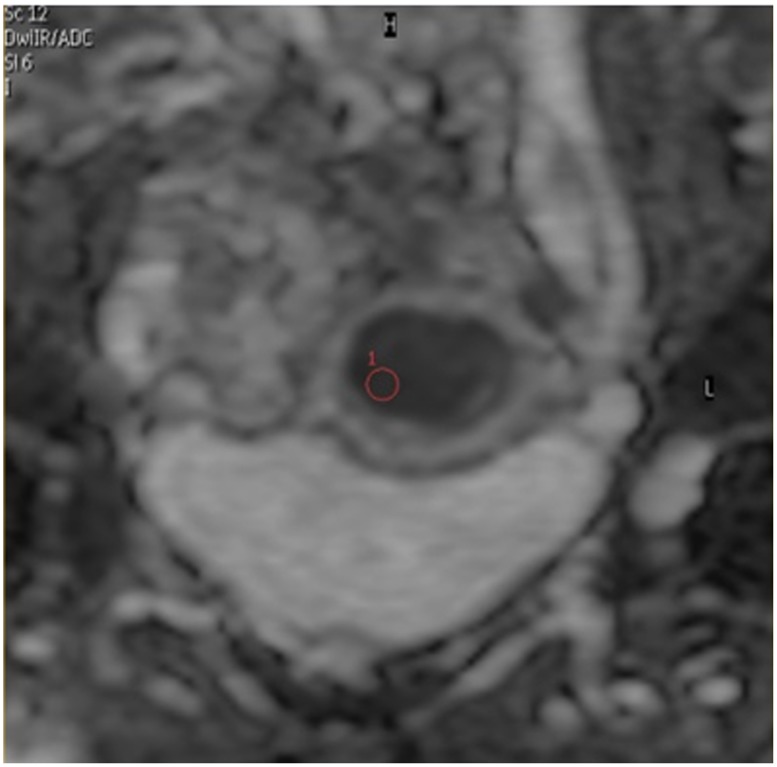
Apparent diffusion coefficient (ADC) analyses of endometrial lesions MRI identified a endometrial carcinoma invading the endometrial cavity with intermediate signal intensity on axial oblique T2-weighted imaging **(A)** and high signal intensity on diffusion weighted imaging (DWI) **(B)**. A region of interest (59.3 mm^2^) was manually defined within the smallest ADC area of the endometrial lesion on a DW-ADC image **(C)**.

ADC maps were generated on the scanner console using axial oblique DW b = 1000 and b = 0 images. For each patient, a region of interest (ROI: 11.9-59.3 mm^2^; mean 49.2 ± 16.9 mm^2^) was manually defined by two radiologists (with 30 years of experience in gynecological MRI) to be as large as possible (max 59.3 mm^2^) within the smallest ADC area of endometrial lesion visualized on DW imaging (b = 0). Care was taken to avoid contamination of this ROI by adjacent normal endometrium or myometrium or by areas of fluid/necrosis within the endometrial cavity (Figure 4). The ROI was determined after discussion between two radiologists. The mean, minimum and maximum ADC values for each tumor grade were compared using a one-way analysis of variance (ANOVA). A ROC curve was generated to evaluate the diagnostic accuracy with the tumor grade as a predictor for DWI. The cut-off point for the ROC was predetermined by JMP software in order to automatically minimize the mathematical distance between the ROC curve and the ideal point, as a method to minimize any misclassification of the tumor grade. The ADC_mean_, ADC_min_ and ADC_max_ (mm^2^/sec) were calculated using the software program for the Philips workstation (Extended MR Workspace; Philips Healthcare, Best, The Netherlands).

### Histopathology

We performed the FS analysis using the following procedure: After hysterectomy, the uterine anterior wall was sectioned through the cervical canal and the endometrial cavity longitudinally and then sectioned horizontally from the fundus to the ostium of the fallopian tube. Gynecologists assessed the deepest point of invasion and oversaw the selection of sections for freezing. One full-thickness section of an endometrial tumor, grossly assessed to be deepest point of invasion, with underlying myometrium and serosa was frozen in Optimal Cutting Temperature media and sectioned on a cryostat into 5-μm slices. Five different levels were cut, separated by approximately 25-50 μm, which were then stained with rapid hematoxylin and eosin staining for microscopy.

Both the microscopic depth of myometrial invasion as well as the tumor subtype and grade were determined on the single frozen section. Occasionally, an additional section was frozen when the first slides showed equivocal findings or the sections were of a particularly poor quality. The microscopic diagnosis was performed by two pathologists. In most cases, the frozen section remnant was then submitted for permanent histology, and several additional sections of tumor were paraffin-embedded and used for the final microscopic analysis. The same pathologists verified the final pathology report. When the pathologists made a different diagnosis, discussions were held until a consensus was reached.

### True positive high-grade tumors and true negative low-grade tumors

The true positive rate of high-grade tumors was defined as the total number of high-grade tumors on pre-operative DW-MRI or intra-operative FSs divided by the total number of high-grade tumors on final pathology. The true negative rate of low-grade tumors was defined as the total number of low-grade tumors on pre-operative DW-MRI or intra-operative FSs divided by the total number of low-grade tumors on final pathology.

### Statistical analyses

All of the statistical analyses were performed using the JMP software package (version. 13.1.0; SAS institution Japan, Tokyo, Japan). The diagnostic agreement between the DWI, preoperative biopsy or FS findings and the final pathology was calculated using kappa statistics. Continuous variables are expressed as the mean ± standard deviation (SD). The Mann-Whitney U-test was used to compare continuous variables between the two groups. Tukey’s honestly significant difference (HSD) was used for making multiple comparisons in datasets with continuous variables containing more than two groups. Fisher’s exact test was used to compare frequencies. P values of <0.05 were considered to indicate statistical significance.

## SUPPLEMENTARY MATERIALS TABLE



## Abbreviations

ADC: apparent diffusion coefficient
FS: frozen section
FIGO: International Federation of Gynecology and Obstetrics
DW-MRI: diffusion-weighted magnetic resonance imaging
PLND: pelvic lymph node dissection
ADC: apparent diffusion coefficient
BMI: body mass index
AUC: area under the curve
ROC: receiver operating characteristic
PS: paraffin sections
TSE: turbo spin echo
SPAIR: spectral attenuated inversion recovery
VISTA: volume isotropic TSE acquisition
3DG: 3-dimensional gradient-echo
TR: repetition time
TE: echo time
FOV: field of view
DCE: Dynamic contrast material-enhanced
ROI: region of interest
ANOVA: analysis of variance
SD: standard deviation
HSD: honestly significant difference
