# Supplementary material for: Preoperative diffusion-weighted magnetic resonance imaging and intraoperative frozen sections for predicting the tumor grade in endometrioid endometrial cancer
Source: Oncotarget. 2018 Nov 27;9(93):36575–84. doi: 10.18632/oncotarget.26366 (PMC6290960; doi:10.18632/oncotarget.26366)
Supplement: Supplementary file 1 [file oncotarget-09-36575-s001.pdf]

Preoperative diffusion-weighted magnetic resonance imaging and intraoperative frozen sections for predicting the tumor grade in endometrioid endometrial cancer

SUPPLEMENTARY MATERIALS

Supplementary Table 1: The magnetic resonance imaging parameters

	Axial T1 TSE	Axial T1 SPAIR	Sagittal and oblique T2 TSE	T2 VISTA	Axial oblique T2 TSE	Axial and axial oblique DWI	3DG T1 SPAIR
TR/TE (msec)	449-500/9	534-757/13	3200-4500/100	3183-5541/147	3200-4500/100	1600-3200/70	4.17/1.98
Section thickness/ slice gap (mm)	5/1	5/1		0.9/0	5/1	5/1	2/0
FOV (mm)	250	250	250	250	250	360	256
matrix	246×352	288×201	265×384	255×256	279×400	144×112	240×240
b vales						1000	

Abbreviations: TSE, turbo spin echo; SPAIR, spectral attenuated inversion recovery; VISTA, volume isotropic TSE acquisition; DWI, diffusion weighted image; 3DG, 3-dimensional gradient-echo; TR, repetition time; TE, echo time; FOV, field of view;
